# Effects on HbA1c of referral of type 2 diabetes patients to secondary care

**DOI:** 10.1080/02813432.2024.2433107

**Published:** 2024-12-01

**Authors:** Kjersti Nøkleby, Anne K. Jenum, Esben Selmer Buhl, Tor Claudi, John G. Cooper, Signe Flottorp, Karianne F. Løvaas, Sverre Sandberg, Tore Julsrud Berg

**Affiliations:** a Department of General Practice, Institute of Health and Society, University of Oslo, Oslo, Norway; b Department of General Practice, Institute of Health and Society, General Practice Research Unit (AFE), University of Oslo, Oslo, Norway; c Department of Medicine, Nordland Hospital, Bodø, Norway; d Norwegian Quality Improvement of Laboratory Examinations, Haraldsplass Deaconess Hospital, Bergen, Norway; e Norwegian Institute of Public Health, Oslo, Norway; f Department of Global Public Health and Primary Care, University of Bergen, Bergen, Norway; g Department of Medical Biochemistry and Pharmacology, Haukeland University Hospital, Bergen, Norway; h Institute of Clinical Medicine, Faculty of Medicine, University of Oslo, Oslo, Norway; i Department of Endocrinology, Morbid Obesity and Preventive Medicine, Oslo University Hospital, Oslo, Norway

**Keywords:** Diabetes mellitus, type 2, referral and consultation, secondary care centres, glycated haemoglobin, general practice

## Abstract

**Aim:**

To study trajectories of HbA1c in type 2 diabetes (T2D) patients referred to diabetes outpatient clinics (DOCs), and to explore characteristics of referrals and patient pathways in patients treated in DOCs.

**Methods:**

We retrospectively followed T2D patients from the Norwegian population-based ROSA 4 study to identify persons with T2D who were referred to a DOC. We used latent class trajectory modelling to identify subgroups of patients with similar patterns of HbA1c one year before to one year after the first consultation at a DOC. We performed multinomial regression analyses to identify baseline characteristics associated with group membership.

**Results:**

Four hundred and two of 6716 T2D patients started treatment at a DOC, constituting a yearly starting rate of 1.5%. We identified three classes of HbA1c trajectories: (1) stable moderate hyperglycaemia (75%); (2) severe hyperglycaemia with a decline in HbA1c around referral (14%) and (3) severe hyperglycaemia with a decline in HbA1c after starting treatment at the DOC (11%). HbA1c trajectories were associated with diabetes duration RRR 0.92, CI (0.87, 0.97) in class 2 vs. 1 and 0.93 (0.88, 0.98) in class 3 vs. 1. Some differences were found between clinics in rejection rate, processes of care, and duration of follow-up.

**Conclusions:**

Norwegian GPs handle most T2D patients themselves. Those with T2D and severe hyperglycaemia had a considerable benefit from being referred to a DOC, though with two separate trajectories: One where HbA1c improved around the time of referral, and another that improved after starting in a DOC.

## Abstract (plain language summary)

The data originate from a high-quality database of type 2 diabetes patients.

The strength of the data-driven latent class trajectory modelling (LCTM) is that it can uncover hidden patterns of HbA1c changes in heterogenic populations.

On the other hand, LCTM have concerns about generalisability, as it may be difficult to replicate.

Among other limitations are a rather small number of patients referred during the study years, increasing the risk of type 2 errors in further analyses.

## Introduction

As type 2 diabetes (T2D) represents an increasing burden for health systems [1], it is important to organise complex health care efficiently and reduce clinical inertia [2].

In many countries, the care of non-communicable diseases including T2D has moved toward general practice [3,4], but the challenge to improve integrated care persists [4,5]. T2D patients with complex disease where the general practitioners (GPs) require a specialist assessment, are most often referred to diabetes outpatient clinics (DOCs), though enhanced primary care or variations of shared care have produced similar clinical outcomes in trials [3,6,7].

In Norway, general practice is the routine level of care for people with T2D [8]. The GP alone generally has the main role in the care and acts as the gatekeeper to secondary care. The national ‘Priority guidelines for secondary care’ recommends a 12-week maximum time for a DOC to see patients with T2D affected by ‘poor metabolic control or late complications, macrovascular disease or other complicating disease’ [9]. No standard clinical pathway in the DOCs exists, implying a large variation in the extent of follow-up.

Few have studied the effect of DOC referrals on glycaemic control in people with T2D [10,11]. Specifically, we are not aware of any studies assessing whether such patients may have different patterns of HbA1c trajectories. Subgroups of persons with T2D where group members had similar trajectories of HbA1c changes have been identified in other settings [12,13], and a similar approach may be employed to investigate the dynamics of glycaemic control among patients with T2D referred to secondary care. This is of importance to allocate resources to the patient group with highest need and best effect.

Therefore, we aimed to study trajectories of HbA1c in patients with T2D referred to DOCs and to explore characteristics of referrals and patient pathways in individuals with T2D treated in DOCs.

## Participants and methods

### Study sample and data sources

Data are drawn from the population-based ROSA 4 study, described in detail elsewhere [14]. In short, it consists of all diabetes patients from 77 practices with 282 GPs, from rural and urban areas in Norway. Research nurses manually verified the diabetes diagnosis and registered complications from the GPs’ electronic medical records (EMRs). Most data from ROSA 4 are cross-sectional from 2014, but laboratory data and prescriptions from the GPs’ EMRs from 2012 to 2014 were available. The linked socioeconomic variables from Statistics Norway are from 2014.

In this sub-study, ROSA 4 patients from two of the four participating regions represent the baseline population: all T2D patients listed with 78 (of a total 78) GPs in the Salten region of northern Norwegian (*n* = 2779) and with 99 out of 161 invited GPs (total 1037 GPs) in the capital region (counties Oslo and Akershus) (*n* = 3937) [15]. These 6716 patients had data to track whether they were referred to a DOC during 2013–2017, but the two cohorts had different sources of data from the DOCs.

**Figure 1.**
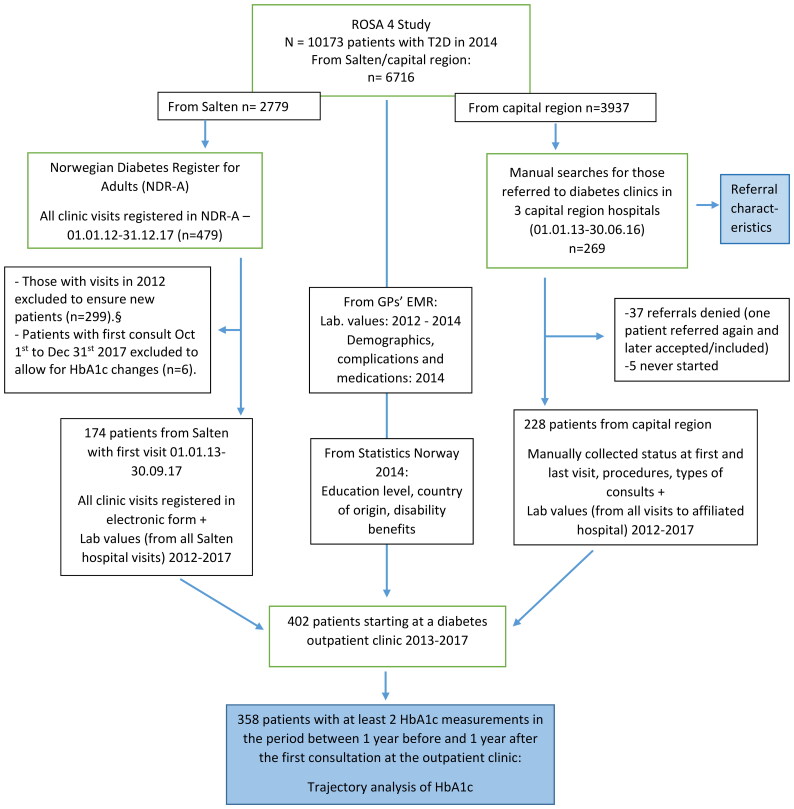
Flowchart of study participants. T2D: type 2 diabetes; EMR: electronic medical record. ^§^From NDR-A/Salten, referrals are not registered, and visits were not marked with the number of the visit, so we had to exclude those who were already under follow-up, who were seen at least yearly. By excluding all patients from Salten registered with a consultation in NDR-A in 2012, this ‘wash-out’ period made sure that everyone having their first registered consultation in 1 January 2013 to 30 September 2017 was starting a period of follow-up at a diabetes outpatient clinic.

In the Salten region, all visits at the DOC (one hospital) were registered in a structured diabetes journal and reported to the Norwegian Diabetes Register for Adults (NDR-A), but not all variables were filled in at each consultation. We have data from laboratory and DOC visits during 2012–2017. We included those having a first consultation between 1 January 2013 and 30 September 2017 (Figure 1).

In the capital region, the districts of the included GP practices belonged to three local hospitals which did not report to NDR-A at the time. Therefore, we first performed a manual search in these hospitals’ EMRs to identify those of the 3937 ROSA 4 patients who were referred to their local DOCs in the period 1 January 2013 to 30 June 2016. Second, we manually collected data both from the referrals and from all hospital follow-ups.

### Covariates

*Laboratory values:* HbA1c, LDL-cholesterol and estimated glomerular filtration rate (eGFR) from GPs (ROSA 4), NDR-A and capital region EMRs (Figure S1).

*Variables from both regional cohorts***:** *From ROSA 4*: Age, sex, diabetes duration and complication status. *From NDR-A/capital region EMRs*: Time of first and last consultation within the follow-up period, number of consultations, group of glucose-lowering medication and blood pressure. *From Statistics Norway*: Education level, country of origin and disability benefits.

*Variables from the capital region only:* Date and source of referral letters, the reason for referral; HbA1c at referral; type of health care professional performing the consultations; glucose-lowering, lipid-lowering, anti-hypertensive and antiplatelet medications at start and end of follow-up at DOCs. Three narrative variables were sorted and categorised: (1) the reason for rejections (GPs should initiate insulin or adjust glucose-lowering medications themselves; insufficient information in referral letter; no need for specialist treatment); (2) advice to GPs in discharge reports (advice on adjustments of glucose-lowering medications; individual HbA1c targets; recommended further investigations; non-specific advice on ‘routine care’; other advice) and (3) advice in reports during follow-up (advice on adjustments of glucose-lowering medications; other advice).

### Statistical analyses

Descriptive statistics are presented as means, medians and frequencies, with one-way ANOVA/Kruskal–Wallis and chi-squared/Fisher’s exact tests to test group differences as appropriate.

We used data-driven latent class trajectory modelling (LCTM) to identify potential subgroups (classes) with similar individual patterns of HbA1c over time [16], using the variables of time, HbA1c and person ID only. Time was centred on the date of the first consultation at the DOC, restricted to one year before and one year after starting treatment in the DOC. We included those with at least two HbA1c measurements during this period in the LCTM analyses (Figure 1).

Using the *hlme* function from the *lcmm* package in RStudio, we identified models with two, three, four and five classes, each with natural cubic splines (with the *Ns* function in the *Epi* package) to allow the HbA1c curve to fluctuate. Given our study group size, we tested models with four and five knots, with a lower Akaike information criterion (AIC) as the selection criterion [17], and found five knots to be optimal. The positioning of the knots was set at the quantiles of time of 0.05, 0.275, 0.5, 0.725 and 0.95 (corresponding to −282, −46, 0, 121 and 316 days) [17].

To identify the model with the optimal number of classes, we evaluated the Bayesian information criterion/AIC, relative entropy, size of the smallest class (a cut-off at minimum 5%), and posterior probability of assignment to each class of the different models (with two, three, four and five classes) (Table S1) [16]. We gave the classes/groups names to describe the trajectories.

We performed multinomial regression analyses (command *mlogit*) to identify baseline patient characteristics associated with class membership, using purposeful selection to build an adjusted model [18]. The class with ‘stable’ HbA1c was used as the reference group as it was the largest.

Statistical analyses were performed with StataSE 16 (StataCorp, College Station, TX) and RStudio Version 1.4.1717 (The R Foundation for Statistical Computing, Vienna, Austria). The significance level was set to .05.

## Results

During the observation period (the Salten region 1 January 2013–31 December 2017; the capital region 1 January 2013–31 December 2016), 402 of the 6716 T2D patients had their first consultation at a DOC (Figure 1), constituting 1.5% of all T2D patients per year. Most patients commencing treatment at a DOC had moderate to severe hyperglycaemia, more foot ulcers and retinopathy and more intensive glucose-lowering treatment, but similar levels of macrovascular complications compared with those not referred (Table S2). The mean HbA1c at the first consultation at the DOC was 8.7% ± 1.8 (72 mmol/mol ± 20), and the mean reduction in HbA1c from starting treatment to the measurement closest to six months after starting at the DOC (from 31 to 365 days, *n* = 288) was 0.8% ± 1.9 (9 mmol/mol ± 21). Thirty-five percent had at least 1.0% (11 mmol/mol) decrease in HbA1c.

### HbA1c trajectories

**Figure 2.**
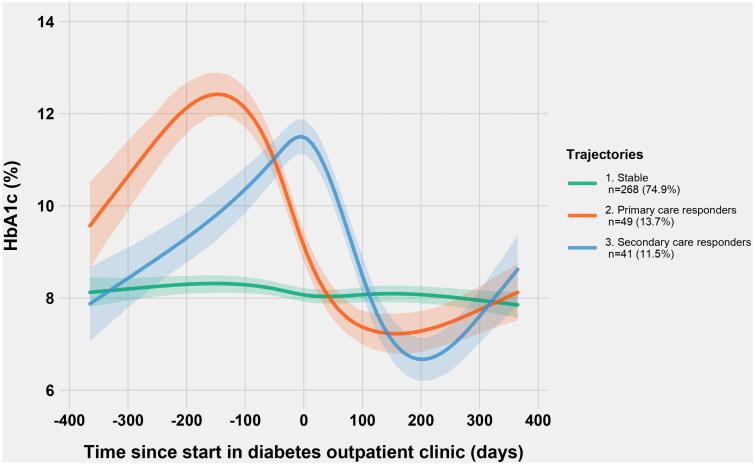
Trajectories of HbA1c (in %) for three latent classes/groups from one year before starting treatment in a diabetes outpatient clinic (at day 0), to one year after. Lines for each group represent the predicted means and 95% confidence limits.

In the LCTM analyses of HbA1c before and after starting treatment at the outpatient clinic, 358 patients were included, and a model with three groups was selected (Figure 2, Table S1). Patients excluded from LCTM analyses because of <2 HbA1c values (*n* = 44) were mostly similar to those included in the trajectory analyses (Table S2), though with shorter follow-up.

The largest group was called ‘stable’ and comprised 75% of the patients. Their HbA1c trajectory showed persistent, moderate hyperglycaemia. The two smaller groups had similar reductions in HbA1c from excessively high values; in ‘primary care responders’ (14%) the reduction started before the first appointment in the DOC, whereas in ‘secondary care responders’ (11%) the reduction started when commencing DOC treatment. Mean HbA1c in ‘primary care responders’ fell from 11.6% (103 mmol/mol) at referral (only values from the capital region) to 9.0% (75 mmol/mol) at the first consultation and further to 7.6% (60 mmol/mol) in the measurement closest to six months after start. In ‘secondary care responders’, HbA1c fell from 12.1% (109 mmol/mol) at referral (from the capital region) to 11.7% (104 mmol/mol) at starting DOC treatment, to 7.4% (57 mmol/mol) after around six months (Table 1).

**Table 1.** Characteristics of the patients with HbA1c trajectories and the three classes in latent class trajectory analysis (*n* = 358).

		Classes/groups from HbA1c trajectory analysis			
	Total	‘Stable’	‘Primary care responders’	‘Secondary care responders’	
*n* (% of total)	*n* = 358	*n* = 268 (74.9%)	*n* = 49 (13.7%)	*n* = 41 (11.5%)	Overall *p*^a^
Age (in 2014) (*n* = 358)	59.2 (13.2)	60.3 (12.8)^b^	55.1 (13.6)	56.4 (14.6)	.014
Diabetes duration (2014) (*n* = 352)	10.9 (8.7)	12.2 (8.9)^b^^,^^c^	7.1 (6.5)	7.5 (7.8)	<.001
Sex (proportion of males) (*n* = 358)	210 (59%)	159 (59%)	31 (63%)	20 (49%)	.345
Level of education (*n* = 333)					.561
Pre- and primary education	109 (33%)	77 (31%)	16 (36%)	16 (41%)	n/a
Secondary education	155 (47%)	120 (48%)	21 (48%)	14 (36%)	n/a
Tertiary education	69 (21%)	53 (21%)	7 (16%)	9 (23%)	n/a
Non-Western country of origin (*n* = 358)^d^	82 (23%)	62 (23%)	12 (24%)	8 (20%)	.842
Disability benefits, proportion if <60 years (*n* = 176)^e^	59 (34%)	42 (34%)	8 (29%)	9 (38%)	.785
Proportion of classes within outpatient clinic (*n* = 358)					0.831
Nordland hospital (Salten region)	163 (46%)	124 (76%)	21 (13%)	18 (11%)	n/a
Oslo University Hospital	73 (20%)	56 (77%)	10 (14%)	7 (9.6%)	n/a
Akershus University Hospital	72 (20%)	50 (69%)	10 (14%)	12 (17%)	n/a
Bærum Hospital	50 (14%)	38 (76%)	8 (16%)	4 (8.0%)	n/a
Main reason for referral (*n* = 195)^f^					.002^g^
Hyperglycaemia	146 (75%)	98 (68%)	25 (89%)	23 (100%)	
Foot ulcer	16 (8.2%)	14 (9.7%)	2 (7.1%)	0	
Other reasons^h^	33 (17%)	32 (22%)	1 (3.6%)	0	
Waiting time from referral to DOC in days (*n* = 174)^f^	56 [22, 91]	63 [24, 98]^b^	50 [22, 65]	31 [4, 56]	.010
Duration of follow-up in days (*n* = 358)	188 [5, 531]	173 [0, 534]	251 [18, 606]	193 [56, 442]	.710
Number of consultations in clinic (*n* = 357)					.075
1–2 consultations	146 (41%)	118 (44%)	18 (37%)	10 (24%)	n/a
3–5 consultations	114 (32%)	82 (31%)	12 (24%)	20 (49%)	n/a
6–10 consultations	65 (18%)	45 (17%)	13 (27%)	7 (17%)	n/a
More than 10 consultations	32 (9.0%)	22 (8.2%)	6 (12%)	4 (10%)	n/a
Types of consultations (proportion) (*n* = 173)^f^					
Doctor	0.40 (0.36)	0.41 (0.36)	0.39 (0.33)	0.32 (0.44)	.609
Diabetes nurse	0.46 (0.39)	0.44 (0.38)	0.46 (0.35)	0.63 (0.45)	.155
Dietitian	0.05 (0.17)	0.06 (0.18)	0.04 (0.10)	0.01 (0.03)	.423
Foot clinic	0.09 (0.26)	0.08 (0.27)	0.11 (0.28)	0.04 (0.18)	.704
Proportion not seeing a doctor during follow-up (*n* = 173)^f^	49 (28%)	35 (27%)	6 (22%)	8 (44%)	.239
*Measurements*					
HbA1c, %/mmol/mol					
At referral (*n* = 174)^f^	9.3 (2.1)/77 (23)	8.4 (1.5)/68 (17)^b^^,^^c^	11.6 (1.7)/103 (19)	12.1 (1.6)/109 (18)	<.001
Nearest first consultation (*n* = 358)^i^	8.7 (1.8)/72 (20)	8.1 (1.3)/65 (14)^b^^,^^c^	9.0 (1.5)/75 (17)^j^	11.7 (1.3)/104 (14)	<.001
Nearest six months after first consult (*n* = 284)^k^	7.9 (1.5)/63 (17)	8.1 (1.6)/61 (18)^c^	7.6 (1.2)/60 (13)	7.4 (1.3)/57 (14)	.011
LDL cholesterol in mmol/l (*n* = 330)^i^	2.7 (1.8)	2.7 (1.0)^c^	2.8 (1.0)	3.3 (1.2)	.007
Systolic blood pressure, mmHg (*n* = 223)^l^	134 (18)	134 (20)	136 (20)	134 (14)	.462
*Complications*					
Estimated GFR, mean (ml/min/1.73 m^2^) (*n* = 355)	84 (25)	83 (25)	85 (26)	92 (22)	.083
Foot ulcer and/or amputation (*n* = 356)	37 (10%)	30 (11%)	6 (12%)	1 (2.4%)	.481
Retinopathy (*n* = 282)	55 (20%)	48 (22%)	6 (18%)	1 (3.6%)	.155
Any macrovascular complication (*n* = 358)^m^	103 (29%)	84 (31%)	10 (20%)	9 (22%)	.177
*Medications changes during follow-up*					
Changes in number of medications (*n* = 191)^f^					.004
1 or 2 less (proportion)	19 (10%)	11 (7.8%)^c^	2 (7.4%)	6 (26%)	
Unchanged	113 (59%)	92 (65%)^c^	15 (56%)	6 (26%)	
1 or 2 more	59 (31%)	38 (27%)	10 (37%)	11 (48%)	
Insulin initiation during follow-up (*n* = 351)	40 (11%)	28 (11%)	3 (6.3%)	9 (22%)	.071

n/a: not applicable with post hoc test.

Data are presented as mean (standard deviation), median [quartile 1, quartile 3] and *n* (%).

^a^ Differences between groups calculated with one-way ANOVA in continuous variables (Kruskal–Wallis when not normally distributed), Chi-square test in categorical variables (Fisher’s exact test when a cell had less than 5 expected persons), with a null hypothesis that there was no difference between the three classes.

^b^ Post hoc pairwise comparison (with correction for multiple testing with Bonferroni) is significant between ‘stable’ and ‘primary care responders’, *p* < .05.

^c^ Post hoc pairwise comparison (with correction for multiple testing with Bonferroni) is significant between ‘stable’ and ‘secondary care responders’, *p* < .05.

^d^ Country of origin other than Western Europe and Northern America.

^e^ Permanent disability benefits and work assessment allowance.

^f^ Only data from capital region.

^g^ Overall result from Fisher’s exact test. Because of some cells with zeros, we performed Barnard’s tests in 2 × 2 contingency tables, which were statistically insignificant except for the comparison between hyperglycaemia and other reasons in ‘stable’ vs. ‘primary care responders’.

^h^ Referred to/for diabetes nurse, nutritionist, overweight, driver’s license, foot problems and hypoglycaemia.

^i^ Measurements closest to first consultation (from 30 days before to 30 days after).

^j^ Post hoc pairwise comparison (with correction for multiple testing with Bonferroni) is significant between ‘primary care responders’ and ‘secondary care responders’, *p* < .05.

^k^ Measurements closest to six months after start (from 31 to 365 days).

^l^ First measurement in diabetes outpatient clinic.

^m^ Stroke, coronary heart disease, amputation and arterial surgery.

**Figure 3.**
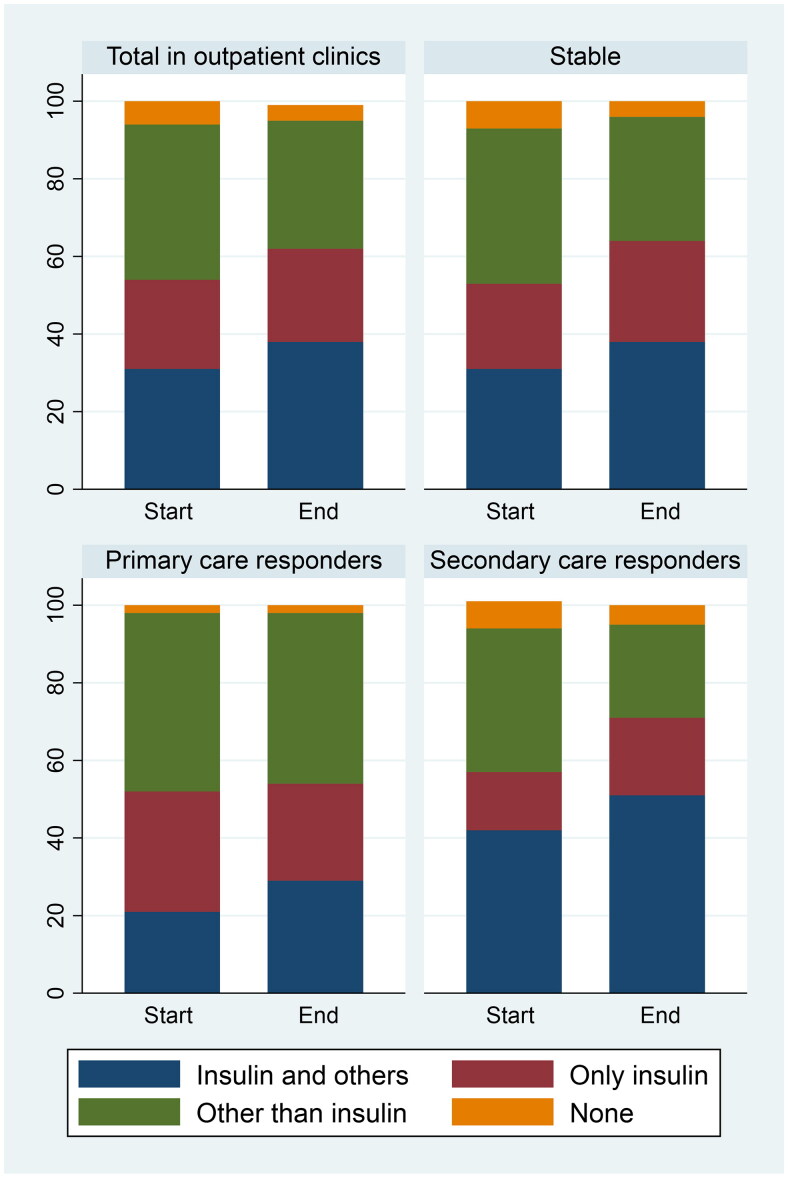
Proportions of groups of glucose-lowering medications, by HbA1c trajectory classes, at the start and end of follow-up at the diabetes outpatient clinics. Total *n* = 402, in ‘stable’ *n* = 268, in ‘primary care responders’ *n* = 49 and in ‘secondary care responders’ *n* = 41.

Compared to the ‘stable’ group, people in both the other HbA1c groups were about five years younger and had shorter diabetes duration (Table 1). All groups had hyperglycaemia as the most common main reason for referral, but the ‘stable’ patients had a larger proportion of other main reasons. The ‘secondary care responders’ had the most changes in medications, accompanied by an abrupt fall in HbA1c after starting treatment at the DOC (Figure 3, Table S4).

One year after starting treatment at the DOCs, the HbA1c trajectories in ‘primary care responders’ and ‘secondary care responders’ showed a slightly increasing HbA1c. However, the total mean HbA1c remained stable around 8.0% (64 mmol/mol) from 9 to 24 months, though the number of persons with measurements was reduced with time.

### Multinomial regression analyses

Among baseline characteristics, only diabetes duration was significantly associated with the HbA1c trajectory groups in the age-adjusted model; with each year’s longer duration entailing a lower risk of being in both ‘primary care responders’ vs. ‘stable’ (RRR 0.92 (95% CI 0.87, 0.97)) and ‘secondary care responders’ compared to ‘stable’ (RRR 0.93 (95% CI 0.88, 0.98)) (Table 2).

**Table 2.** Baseline characteristics associated with HbA1c trajectory class membership in multinomial regression, with class 1, ‘stable’ as reference group.

	‘Primary care responders’ vs. ‘stable’				‘Secondary care responders’ vs. ‘stable’			
	Unadjusted univariate		Adjusted model (*n* = 352)		Unadjusted univariate		Adjusted model (*n* = 352)	
Baseline characteristics	RRR	*p* Value	RRR	*p* Value	RRR	*p* Value	RRR	*p* Value
Age in 2014 in years	0.97 (0.95, 0.99)	.011	0.99 (0.96, 1.01)	.378	0.98 (0.95, 1.00)	.072	0.99 (0.97, 1.02)	.689
Sex (reference male)	0.85 (0.45, 1.59)	.605			1.53 (0.79, 2.96)	.205		
Diabetes duration (2014) in years	0.91 (0.87, 0.96)	<.001	0.92 (0.87, 0.97)	.002	0.92 (0.87–0.97)	.002	0.93 (0.88, 0.98)	.006
Level of education^a^								
Pre- and primary education	1.28 (0.66, 2.51)	.465			1.56 (0.78, 3.12)	.206		
Country of origin^b^								
Non-Western	1.08 (0.53, 2.19)	.837			0.81 (0.35, 1.83)	.606		
Any macrovascular complication (reference none)	0.56 (0.27, 1.18)	.127			0.62 (0.28, 1.35)	.225		
Region (reference capital region)	1.15 (0.62, 2.13)	.660			1.10 (0.57, 2.13)	.777		

Presented as RRR (upper limit, lower limit): relative risk ratio with 95% confidence intervals. The adjusted model is adjusted for age and diabetes duration, after purposeful model selection. *N* = 358 for univariate models, except diabetes duration (*n* = 352) and education (*n* = 333).

^a^ *Reference*: Secondary or tertiary education.

^b^ *Reference*: Norway, Western Europe and North America.

### Characteristics of referrals and those rejected in the capital region

Most (67%) were referred by a GP and 29% by other hospital departments, with hyperglycaemia being the most common main reason for referral (73%) (Table S3). Foot ulcers were the main reason for referral in 11%, whereas the remaining 16% were referred for an appointment to see a diabetes nurse or nutritionist, or for overweight, driver’s license, other foot problems or hypoglycaemia. The median waiting time between referral and first consultation at the DOC was 56 days.

Overall, 14% of referrals in the capital region were rejected, with substantial variation between the three clinics (7.5%, 7.7% and 23%, respectively). Mean HbA1c at referral among those accepted was 9.3% (78 mmol/mol) and 8.8% (73 mmol/mol) in those rejected. Most rejection letters (68%) advised the GPs to initiate insulin or to adjust glucose-lowering medications themselves. Insufficient information in the referral letter, or ‘no need for specialist treatment’ was stated as the reason for the remaining rejections. Akershus University Hospital, which rejected the largest proportion of referrals, had a standard rejection letter stating the responsibilities of the GPs in treating and educating T2D patients, including an algorithm on how to initiate insulin, often adding specific advice.

### Patient pathways

The median number of consultations was three (range 1–54), and the median follow-up time was 138 days, with marked differences between the clinics (lowest median 62, highest 285) (Table S3). Figure S2 shows the proportions of processes of care performed during follow-up in the DOCs stratified by regions. Foot examination and albuminuria measurements were performed least, with regional differences: in the capital region 48% had their urine examined, compared to 70% in Salten. During follow-up, 10% of those commencing DOC treatment, initiated insulin. Table S4 shows more details about medication changes.

Overall, in the capital region, nurses had the largest proportion of consultations (46%), while physicians had 40% of consultations (Table 1), and 28% of patients never had a consultation with a physician during DOC follow-up.

In the discharge reports for the capital region patients, specific advice to primary care providers was given in 29%. Mostly, advice was given about recommended insulin adjustments or possible next-step glucose-lowering medications, but also regarding individual HbA1c targets, recommended further investigations or treatment for neuropathic pain. Instead of specific advice, many GPs were advised to follow ‘routine care’ or to adhere to the national clinical guidelines, but also to refer again or to call an endocrinologist on duty if there were questions. As some reports to the GPs during follow-up also contained advice (mostly about adjustments of glucose-lowering medications), a total of 45% of patients had advice given to their GP during follow-up or at discharge.

## Discussion

To the best of our knowledge, the present retrospective cohort study is the first to analyse trajectories of HbA1c changes in patients with T2D referred to DOCs. Among the 1.5% who started treatment at a DOC each year, we identified three groups with different HbA1c trajectories, where the largest group (‘stable’) with longer diabetes duration, had moderate, persistent hyperglycaemia both before and after starting treatment at the DOC. The two smaller groups had more severe hyperglycaemia, followed by a reduction in HbA1c; the ‘primary care responders’ with the peak around referral, and the ‘secondary care responders’ at the time of the first consultation.

Few studies have examined changes in HbA1c after starting treatment in secondary care. A Canadian study reported a reduction in mean HbA1c from 8.8% (73 mmol/mol) to 7.8% (62 mmol/mol) at both 6 and 12 months [10], while a UK study reported a reduction from 9.8% (84 mmol/mol) to 8.8% (73 mmol/mol) at the end of follow-up in secondary care [11]. This is in line with our finding of a mean reduction of 0.8% (9 mmol/mol) from 8.7% (72 mmol/mol).

We found a low annual rate of patients with T2D starting follow-up in secondary care (1.5%), indicating that Norwegian GPs handle most patients themselves. Two other studies report the referral rate, which might be slightly larger due to rejections and no-shows: a Welsh study in 2017/18 found a yearly referral rate to secondary care of 4.2% for both diabetes types [19], while a Dutch study had a 5.3% yearly referral rate of patients with T2D to internists in 2004 and 2006 [20].

Interestingly, HbA1c among the ‘primary care responders’ was reduced before the first consultation at the DOC, in line with our clinical experience that glucose control improves in some patients between referral and the first appointment. We can only speculate about possible reasons for this. Effective communication in primary care about hospital referrals can help patients understand the seriousness of their situation and increase their adherence to lifestyle and medication advice. The GPs may also have adjusted the medications around the time of the referral. Somewhat surprisingly, ‘primary care responders’ did not have fewer consultations at the DOC than the other groups. The reason for this is unknown, but one hypothesis is that experiencing better glycaemic control might lead to a high patient motivation for further improvements.

The ‘secondary care responders’ also had a substantial and clinically important decrease in HbA1c (on average 4.3% (47 mmol/mol)) after starting treatment at a DOC, and most changes in medications. Some of these patients might have benefitted from an earlier referral or earlier insulin initiation by their GPs [21,22], thus representing GP clinical inertia. However, many barriers to insulin initiation exist, for example, patients’ fear or GPs’ lack of technical know-how [23]. Thus, our findings indicate that a referral to secondary care might be ‘two interventions in one’ – first the communication (and possibly measures) around the time of referral, and second the interventions initiated by the DOC.

DOC follow-up had no obvious effects on glycaemia in the large ‘stable’ group. The ‘stable’ group had longer diabetes duration and higher age, and around 30% had other main reasons for referral than hyperglycaemia. However, with a mean HbA1c at the first consultation of 8.1% (65 mmol/mol), hyperglycaemia was still the most common main reason for referral. One could question whether these had any benefits from DOC treatment, but we lack data to investigate this thoroughly. With older patients with longer diabetes duration, individualised HbA1c targets could be around 8% (64 mmol/mol) or even higher in some of these patients [24], and the GPs possibly wanted a second opinion about individual treatment targets. Some alterations in medication were performed without any impact on mean HbA1c, which might reflect problems with hypoglycaemia.

In both groups with severe hyperglycaemia the mean HbA1c levels increased slightly at the end of the first year after starting at a DOC. This is in line with intervention studies [25], probably reflecting the progressive nature of T2D [26] and a declining effect after ending a behavioural intervention [25]. However, an improvement from severe hyperglycaemia in these relatively young patients with a high risk of vascular complications may be clinically important even if transient. From around one to two years after starting at the DOC, however, the mean HbA1c level remained quite stable. As the number of patients with measurements decreased with time, the lasting effect of the DOC intervention might be underestimated due to a possible inherent bias that those with the longest follow-up were those whose hyperglycaemia was most difficult to reduce.

The HbA1c of both the primary care responders and the secondary care responders is substantially lower one year before start at the diabetes outpatient clinics than at the peak values. This could represent the referred patients having a rather fast emerging hyperglycaemia due to poorer life style, less adherence of medications, or loss of insulin production. However, as the trajectories are based on fewer HbA1c values the further away they were from the day of starting treatment in DOCs, this is a more uncertain finding than the abrupt decrease in HbA1c in these groups.

In the capital region, we found large differences in rejection rates between hospitals, where one DOC to a larger extent placed the responsibility for initiating insulin treatment with the GPs, possibly due to capacity problems. Similarly, the differences in the duration of follow-up and the number of consultations may result from differences in the use of resources or local policies.

The DOCs seemed to have a primarily glycaemic focus as hardly any changes in the use of statins or antihypertensives were observed, and the rates of screening for microvascular complications and recordings of weight and BP were unsatisfactory. With screening of albuminuria performed in less than half of patients in the capital region, opportunities for improved treatment and prognosis might have been lost [27]. Differences between regions in the performance of processes of care might reflect that, unlike the capital clinics, the clinic in Salten used a structured follow-up form that has earlier been shown to be associated with GPs’ processes of care [28].

The major strength of this study is the detailed high-quality database of T2D patients considered representative of Norway [14], including laboratory data from both primary and secondary care. The high-quality data collected manually from the DOCs in the capital region were even more detailed, allowing us to describe the clinical pathways in these DOCs. Regarding the HbA1c changes, the strength of the data-driven LCTM is that it can uncover hidden patterns in heterogenic populations.

However, limitations are a rather small number of patients referred, leading to small responder groups, which increases the risk of type 2 errors in further analyses. A lack of power also hindered other relevant subgroup analyses, such as stratification by the reason of referral. Moreover, details of the medications, referrals and clinical pathways were lacking in the Salten data. Furthermore, the HbA1c measurements were highly unbalanced with respect to time, making it difficult to calculate HbA1c changes at given time points. However, this is not a problem in fitting a LCTM, but as this method is entirely data-dependent, it may be difficult to replicate. We did not have data on weight changes, adherence to medications, and lifestyle treatments, nor on any GP interventions around the time of referral. HbA1c measurements following discharge from the DOCs were mainly lacking, which is unfortunate for those with a very short period of follow-up, leading to more patients with <2 HbA1c measurements which gave less power and a possible selection bias in trajectory analyses.

As both groups with severe hyperglycaemia had very high HbA1c before the values fell, some regression to the mean may have been operating [29,30]. On the other hand, these groups are of high risk of developing vascular complications. HbA1c trajectories from those not referred would have been interesting for comparison, but outside the scope of this article and limited by the data. As follow-up ended in 2017, newer diabetes medications were used less than today. An increased use of GLP1-analogues and SGLT2-inhibitors in primary care might have reduced the need for treatment in secondary care. Furthermore, these medications have probably changed medical treatment in the DOCs as well. However, we presume that the same mechanisms that led some patients to reduce their HbA1c before starting at a DOC, apply to other referral settings as well.

## Conclusions

By clustering the individual HbA1c trajectories of patients with T2D referred to DOCs into three groups, we found that patients with severe hyperglycaemia had a considerable benefit from being referred. One subgroup had improved before starting DOC treatment, illustrating a phenomenon of change where psychological mechanisms may be in play and where treatment in primary care may have a larger potential.

## Supplementary Material

Supplemental Material
