# Supplementary material for: Effects on HbA1c of referral of type 2 diabetes patients to secondary care
Source: Scand J Prim Health Care. 2024 Dec 1;43(2):313–23. doi: 10.1080/02813432.2024.2433107 (PMC12090250; doi:10.1080/02813432.2024.2433107)
Supplement: Supplemental Material [file IPRI_A_2433107_SM6181.docx]

Supplementary information: Tables S1-S4

Table S1. Model selection: Statistical characteristics of different trajectory models (2-class, 3-class, 4-class, 5-class)

|  | BIC | AIC | Relative entropyᵃ | Mean APPAᵇ | Size of smallest class;  n (%) |
| --- | --- | --- | --- | --- | --- |
| 2 classes | 6663.153 | 6604.945 | 0.826 | 0.928 | 60 (16.8%) |
| 3 classes* | 6506.189 | 6424.698 | 0.810 | 0.888 | 41 (11.5%) |
| 4 classes | 6417.972 | 6313.198 | 0.829 | 0.880 | 13 (3.6%) |
| 5 classes | 6305.231 | 6177.173 | 0.817 | 0.875 | 16 (4.5%) |
| Recommendation: | Smallest | Smallest | Close to 1 | Greater than 0.70 | Cut-off with |
|  | possible | possible |  | for each class | smallest group at 5% |

AIC= Akaike information criterion, BIC= Bayesian information criterion. ᵃ Values close to 1 indicate lowest classification uncertainty. ᵇ APPA: Average Posterior Probability Assignment: looks at whether individuals are assigned to a class with a high probability and the overall average probability of assignment to each class. *In the selected 3-class model, APPA is 0.938, 0.847 and 0.879 in class 1, 2 and 3 respectively.

Table S2: Descriptive characteristics of type 2 diabetes patients in different samples: the total sample (n= 6716), those followed only in primary care (n=6138), those starting at a diabetes outpatient clinic (DOC) (n=402), those excluded from trajectory analyses (n=44) and those included in trajectory analysis (n=358).

|  |  | Total sample: | T2D patients | Total started in | Started in DOC | Started in DOC |
| --- | --- | --- | --- | --- | --- | --- |
|  |  | T2D patients from | only treated | outpatient clinic | but not in | and in |
|  |  | capital region & Salten | in primary careᵃ |  | trajectory analysisᵇ | trajectory analyses |
| n |  | n= 6716 | n= 6138 | n=402 | n=44 | n=358 |
| Age in 2014 (n= 6716) | | 64.8 (13.1) | 65.3 (12.9) | 59.6 (13.2) | 63.5 (12.7) | 59.2 (13.2) |
| Diabetes duration in years (2014) (n=6284) | | 8.7 (6.9) | 8.4 (6.7) | 10.5 (8.0) | 11.3 (5.4) | 10.9 (8.7) |
| Sex (proportion of males) (n=6716) | | 3634 (54%) | 3295 (54%) | 232 (58%) | 22 (50%) | 210 (59%) |
| Proportion with pre- and primary education (n=6571) | | 2416 (37%) | 2225 (37%) | 137 (35%) | 15 (36%) | 109 (33%) |
| Non-Western country of origin (n=6716)ᶜ | | 1319 (20%) | 1198 (20%) | 96 (24%) | 14 (32%) | 82 (23%) |
| **Measurements** | |  |  |  |  |  |
| HbA1c (n=6509)ᵈ | |  |  |  |  |  |
|  | HbA1c in % | 7.1 (1.2) | 7.0 (1.2) | 8.7 (1.8) | 8.8 (2.0) | 8.7 (1.8) |
|  | HbA1c mmol/mol | 54 (13) | 53 (13) | 72 (20) | 73 (22) | 72 (20) |
|  | Proportion with HbA1c≥8% (64 mmol/mol) | 1254 (19%) | 1011 (17%) | 250 (62%) | 25 (58%) | 225 (63%) |
|  | Proportion with HbA1c≥9% (75 mmol/mol) | 533 (8.2%) | 406 (6.8%) | 153 (38%) | 14 (33%) | 139 (39%) |
| LDL cholesterol in mmol/l (n= 5692)ᵈ | | 2.7 (0.9) | 2.7 (0.90) | 2.7 (1.1) | 2.5 (1.0) | 2.7 (1.1) |
| Systolic blood pressure in mmHg (n=5790)ᵈ | | 135 (17) | 136 (17) | 135 (15) | 140 (14) | 134 (15) |
| Estimated GFR (ml/min/1.73²) (n= 6346)ᵈ | | 82 (21) | 82 (21) | 86 (25) | 84 (22) | 86 (26) |
| **Complications and medications** | |  |  |  |  |  |
| Foot ulcer or amputation (n=6711) | | 216 (3.2%) | 156 (2.5%) | 49 (12%) | 12 (28%) | 37 (10%) |
| Retinopathy (n=3854) | | 481 (12%) | 386 (11.2%) | 57 (18%) | 2 (6.9%) | 55 (20%) |
| Any macrovascular complication (n=6686)ᵉ | | 1841 (28%) | 1676 (27%) | 117 (29%) | 14 (32%) | 103 (29%) |
| Proportion using insulin (n=6716)ᶠ | | 1103 (16%) | 833 (14%) | 213 (54%) | 24 (59%) | 189 (54%) |

Data are presented as mean ± standard deviation, median [quartile 1, quartile 3], n (%). T2D: Type 2 diabetes, DOC: Diabetes outpatient clinic, GFR: Glomerular filtration rate. ᵃ The group in primary care: n=6138. The total sample except 402 starting in diabetes outpatient clinic and 176 with main follow-up already in specialist care in 2013/14 (at least two consultations/year). ᵇ Those starting in a diabetes outpatient clinic with <2 HbA1c measurements were not included in latent class trajectory analyses. ᶜ Non-western origin: country of origin other than Western Europe and North America. ᵈ In patients treated only in primary care: last measurement before 31.12.14. For those starting in diabetes outpatient clinics: nearest first consultation in specialist clinic. ᵉ Composite macrovascular variable: any of coronary heart disease, stroke, amputation, arterial surgery. ᶠ For primary care patients: treatment at 31.12.2014 (prescriptions last 15 months). For those starting in a diabetes outpatient clinic: medication at time of first consultation.

Table S3. Characteristics of referrals and patient pathways in diabetes outpatient clinics, stratified by clinic (n=402)

|  |  |  | Oslo | Akershus | Bærum | Nordland |
| --- | --- | --- | --- | --- | --- | --- |
|  |  |  | University | University | hospital | hospitalᵃ |
| Diabetes outpatient clinic | | Total | Hospital | Hospital |  |  |
| Number of patients with follow-up | | 402 | 80 | 88 | 60 | 174 |
| Duration of follow-up in days (n=402) | | 138 [0, 491] | 62 [8, 316] | 91 [0, 329]ᵇ | 199.5 [31, 535] | 285 [0, 768]ᵇ |
| Number of consultations (n=402) | | 3 [1, 6] | 3 [2,6] | 2 [1, 4] | 4 [2,7] | 3 [1, 6] |
| Waiting time (days from referral to start) (n=175) | | 56 [22, 91] | 56 [23.5, 86] | 53 [14, 89] | 57.5 [19.5, 95.5] |  |
| **Referral characteristics from the capital region** | |  |  |  |  |  |
| Number of referrals | | 269 | 94 | 110 | 65 |  |
| Referrer (n=269) | |  |  |  |  |  |
|  | General practitioner | 179 (67%) | 57 (61%) | 82 (75%) | 40 (62%) |  |
|  | Hospital (ward or other outpatient clinic) | 78 (29%) | 35 (37%) | 21 (19%) | 22 (34%) |  |
|  | Others | 12 (4.4%) | 2 (2.1%) | 7 (6.4%) | 3 (4.6%) |  |
| Main reason for referral (n=269) | |  |  |  |  |  |
|  | Hyperglycemia | 196 (73%) | 67 (71%) | 83 (75%) | 46 (71%) |  |
|  | Foot ulcer | 30 (11%) | 11 (12%) | 11 (10%) | 8 (12%) |  |
|  | Other reasonsᶜ | 42 (16%) | 16 (17%) | 16 (15%) | 11 (17%) |  |
| Referred before 2013 also (n=267) | | 89 (33%) | 28 (29%) | 33 (31%) | 28 (44%) |  |
| Referral rejected (n=268) | | 37 (14%) | 7 (7.5%) | 25 (23%) | 5 (7.7%) |  |
| HbA1c from referral letter (n=228)ᵈ | | 9.3 (2.1)/ 78 (23) | 9.1 (2.1)/ 76 (23) | 9.4 (2.4)/ 79 (26) | 9.3 (1.7)/ 78 (19) |  |
| HbA1c by reason for referral (n=228)ᵈᵉ | |  |  |  |  |  |
|  | Hyperglycemia | 9.9 (2.0)/ 85 (22) | 9.5 (2.0)/ 80 (22) | 10.2 (2.2)/ 88 (24) | 9.7 (1.4)/ 83 (15) |  |
|  | Foot ulcer | 7.8 (1.5)/ 62 (16) | 8.8 (1.3)/ 73 (14) | 7.1 (1.4)/ 54 (15) | 6.2 (-)/ 44 (-) |  |
|  | Othersᶜ | 7.0 (1.0) / 53 (11) | 6.9 (0.7)/ 52 (8) | 6.7 (0.7)/ 50 (8) | 7.5 (1.6)/ 58 (17) |  |
| HbA1c from referral, by referrer (n=228)ᵈ | |  |  |  |  |  |
|  | General practitioner | 9.2 (1.9)/ 77 (21) | 9.3 (2.0)/ 78 (22) | 9.2 (2.1)/ 77 (23) | 9.1 (1.5)/ 76 (16) |  |
|  | Hospital or others | 9.5 (2.4)/ 80 (26) | 8.8 (2.2)/ 73 (24) | 10.0 (2.3)/ 86 (25) | 9.7 (2.1)/ 83 (23) |  |

Data are presented as mean (standard deviation), median [interquartile range] and number (proportion in %). ᵃFrom Nordland hospital (Salten region) we had no data on referrals. ᵇ 0 (zero) as 25% percentile means that at least 25% ended follow-up the same day as the first consultation. ᶜ Referred to/for diabetes nurse, nutrionist, overweight, driver’s license, foot problems, hypoglycemia. ᵈ HbA1c in %/mmol/mol. ᵉ From referral letter.

Table S4. Medication changes in the diabetes outpatient clinics in the capital region.

|  |  | Start of treatment | End of follow-up |
| --- | --- | --- | --- |
| Number of glucose-lowering medications (n=226) | |  |  |
|  | 0 | 16 (7.1%) | 9 (4.0%) |
|  | 1 | 59 (26%) | 51 (23%) |
|  | 2 | 89 (39%) | 83 (37%) |
|  | 3 | 53 (23%) | 64 (28%) |
|  | 4-5 | 9 (4.0%) | 18 (8.0%) |
| Mean number of glucose-lowering medications (n=226) | | 1.92 (0.98) | 2.14 (0.99) |
| Specific glucose-lowering medications | |  |  |
|  | Intermediate-acting insulin (n=226) | 102 (45%) | 122 (54%) |
|  | Longacting insulin (n=226) | 11 (4.9%) | 21 (9.3%) |
|  | Short-acting insulin (n=226) | 51 (23%) | 70 (31%) |
|  | Total insulin dose, international units (n= 107/137) | 50 [30, 88] | 46 [28, 80] |
|  | Metformin (n=226) | 151 (67%) | 147 (65%) |
|  | Sylfonylurea (n=226) | 41 (18%) | 27 (12%) |
|  | SGLT2-inhibitor (n=226) | 8 (3.5%) | 17 (7.6%) |
|  | GLP1 agonist (n=226) | 16 (7.1%) | 28 (12%) |
|  | DPP4-inhibitor (n=226) | 57 (25%) | 54 (24%) |
| Lipid-lowering medication (n=221) | | 124 (56%) | 124 (56%) |
| ACE inhibitor or AT2-blocker (n=221/218) | | 104 (47%) | 109 (50%) |
| Antiplatetelet medication (n=220/217) | | 85 (39%) | 80 (37%) |
| Number of antihypertensive medications | | 1.22 (1.23) | 1.27 (1.22) |

Data are presented as n (%), mean (standard deviation), median [quartile 1, quartile 3]. SGLT2-inhibitor: Sodium-glucose cotransporter-2 inhibitor. GLP1-analogue: Glucagon-like peptide-1 receptor agonist. DPP4 inhibitor: Dipeptidyl Peptidase-4 inhibitors. ACE inhibitor: Angiotensin-converting enzyme inhibitor. AT2-blocker: Angiotensin II receptor antagonist
